# [Retracted] MicroRNA‑758 inhibits tumorous behavior in tongue squamous cell carcinoma by directly targeting metadherin

**DOI:** 10.3892/mmr.2023.13001

**Published:** 2023-04-24

**Authors:** Yulan Zhang, Fuquan Zhao

Mol Med Rep 19: 1883–1890, 2019; DOI: 10.3892/mmr.2019.9805

Following the publication of this paper, it was drawn to the Editor's attention by a concerned reader that certain of the Transwell invasion assay data shown in Fig. 5E were strikingly similar to data appearing in different form in other articles written by different authors at different research institutes, several of which have already been retracted. Owing to the fact that the contentious data in the above article had already been published prior to its submission to *Molecular Medicine Reports*, the Editor has decided that this paper should be retracted from the Journal. After having been in contact with the authors, they accepted the decision to retract the paper. The Editor apologizes to the readership for any inconvenience caused.

